# From uric acid to tophi: multistage molecular and cellular mechanisms of tophi formation

**DOI:** 10.3389/fimmu.2026.1847953

**Published:** 2026-06-10

**Authors:** Heguo Yan, Bo Yang, Niqin Xiao, Jian Zhang, Yundong Xu, Bingbing Chen, Sanjin Zeng, He Qian, Shengyi Zhao, Rong Wang, Jing Xie, Zhaofu Li, Zhaohu Xie

**Affiliations:** 1 Yunnan University of Chinese Medicine, Kunming, Yunnan, China; 2 Zhaotong Hospital of Traditional Chinese Medicine, Zhaotong, Yunnan, China; 3 Yunnan Key Laboratory of Integrated Traditional Chinese and Western Medicine for Chronic Disease in Prevention and Treatment, Kunming, Yunnan, China

**Keywords:** cellular transdifferentiation, granuloma, inflammasome, macrophages, neutrophil extracellular traps, sodium urate crystals, tophi

## Abstract

Tophus is a hallmark lesion of chronic gout, formed through the combined effects of monosodium urate (MSU) crystal deposition, persistent inflammation, and progressive tissue fibrosis. These lesions can cause joint deformities, functional impairment, and renal damage, resulting in a significant decline in patients’ quality of life. Although hyperuricemia has traditionally been regarded as the primary cause of tophus formation, this explanation does not fully account for the considerable clinical heterogeneity observed among patients. In this study, we describe the multistage and dynamic pathological process underlying tophus formation. Building upon the foundation of hyperuricemia, the study focuses on MSU crystal nucleation, growth, and aggregation; crystal-triggered innate immune activation and NLRP3 inflammasome-mediated inflammatory cascades; the dual roles of neutrophil infiltration and neutrophil extracellular traps; macrophage phenotypic conversion, fibroblast activation, and extracellular matrix remodeling; the formation of multinucleated giant cells, complex cellular infiltration, and pathological angiogenesis; and the final formation of a mature, dense fibrous capsule structure. The study identifies the core regulatory nodes at each stage. Additionally, it explores potential therapeutic strategies for tophi and outlines future research directions. Together, these insights provide new therapeutic targets and a more comprehensive conceptual framework for early intervention and drug development. This research carries significant clinical and scientific value, with strong potential to improve outcomes for patients with chronic gout and to reduce the associated healthcare burden.

## 1 Introduction

Gout is a metabolic rheumatic disease caused by abnormalities in purine metabolism or reduced uric acid excretion, with hyperuricemia as the central pathological feature. Clinically, it may present as acute gouty arthritis, chronic gouty arthritis, tophus formation, and gout-related renal damage (1). Among these manifestations, tophi are a defining feature of chronic gout. They consist of monosodium urate (MSU) crystals deposited in periarticular and subcutaneous tissues, together with long-standing inflammation and tissue fibrosis. The appearance of tophi indicates progression to the chronic stage of the disease and can eventually lead to joint deformities, functional impairment, and in severe cases renal failure. These complications greatly reduce the quality of life of affected individuals and contribute to an increased healthcare burden (2, 3). Epidemiological data shows that the global prevalence of gout is between 1 percent and 4 percent, and the incidence continues to rise, with younger individuals increasingly affected. In China, the male-to-female ratio is about 15 to 1, and the frequency of tophus formation increases markedly in patients with a disease duration of more than ten years (4). Despite this, the mechanisms responsible for the development of tophi are still not well understood. The condition has traditionally been explained simply as crystal deposition resulting from persistent hyperuricemia. This explanation, however, does not clarify why many individuals with elevated uric acid levels do not develop tophi (5, 6) and offers limited guidance for developing precise clinical interventions.

With the progression of scientific research, it is now increasingly evident that tophus formation results from multisystem dysregulation involving metabolic pathways, immune responses, coagulation processes, and tissue repair mechanisms, all of which are shaped by complex intercellular communication. Notably, deposition of MSU crystals can even occur during phases of asymptomatic hyperuricemia (7). Current research emphasize the interactions between MSU crystals and immune cells, the formation of neutrophil extracellular traps (NETs), macrophage phenotypic transitions, and the mechanisms underlying fibrosis (8–10). However, several critical questions remain unresolved, including the precise regulation of MSU crystal nucleation, the context-dependent effects of NETs, the key molecular drivers of fibrin capsule formation, and the combined influence of genetic and environmental factors. In light of these gaps, the present study provides a systematic analysis of the molecular and cellular processes involved in each stage of tophus development. It identifies central regulatory nodes within these stages, highlights potential therapeutic targets, and proposes precision-based intervention strategies. The overall aim is to offer new conceptual and mechanistic insights that support early intervention and targeted therapy for tophus formation, ultimately improving outcomes for patients with chronic gout, reducing the clinical and societal burden, and contributing to advances in the diagnosis and treatment of gout.

## 2 Initiation phase: uric acid supersaturation in body fluids and altered physicochemical conditions

### 2.1 Causes of hyperuricemia and its effects on the local microenvironment

The fundamental pathophysiological basis of tophus formation is the persistent elevation of serum uric acid above its solubility threshold, leading to urate supersaturation. Hyperuricemia arises primarily from an imbalance between uric acid production and excretion, which can be broadly classified into decreased uric acid elimination, increased endogenous purine synthesis, or a combination of both. With respect to uric acid excretion, the kidneys are the major regulatory organ, and renal impairment remains the most frequent underlying cause of reduced urate clearance (11). Dysregulation of key transporters is particularly important. Increased activity or expression of urate transporter 1 (URAT1) enhances proximal tubular reabsorption of uric acid (12), whereas loss-of-function variants or reduced expression of the ATP-binding cassette transporter G2 (ABCG2) diminish both renal and intestinal uric acid excretion capacity (13). On the production side, abnormalities in purine metabolism play a central role. Excessive phosphoribosyl pyrophosphate synthetase (PRPS) activity and reduced hypoxanthine-guanine phosphoribosyltransferase (HGPRT) activity contribute to elevated purine synthesis or impaired salvage pathways, promoting hyperuricemia (14). Increased xanthine oxidoreductase (XOR) activity further accelerates the conversion of hypoxanthine and xanthine into uric acid (15). Additionally, deficiency of intestinal farnesoid X receptor (FXR) has been shown to enhance local uric acid production by inducing XOR activation (16). This integrated dysregulation of urate synthesis and excretion forms the biochemical foundation for sustained hyperuricemia, thereby predisposing individuals to MSU crystal formation and subsequent tophus development.

**Figure 1:**
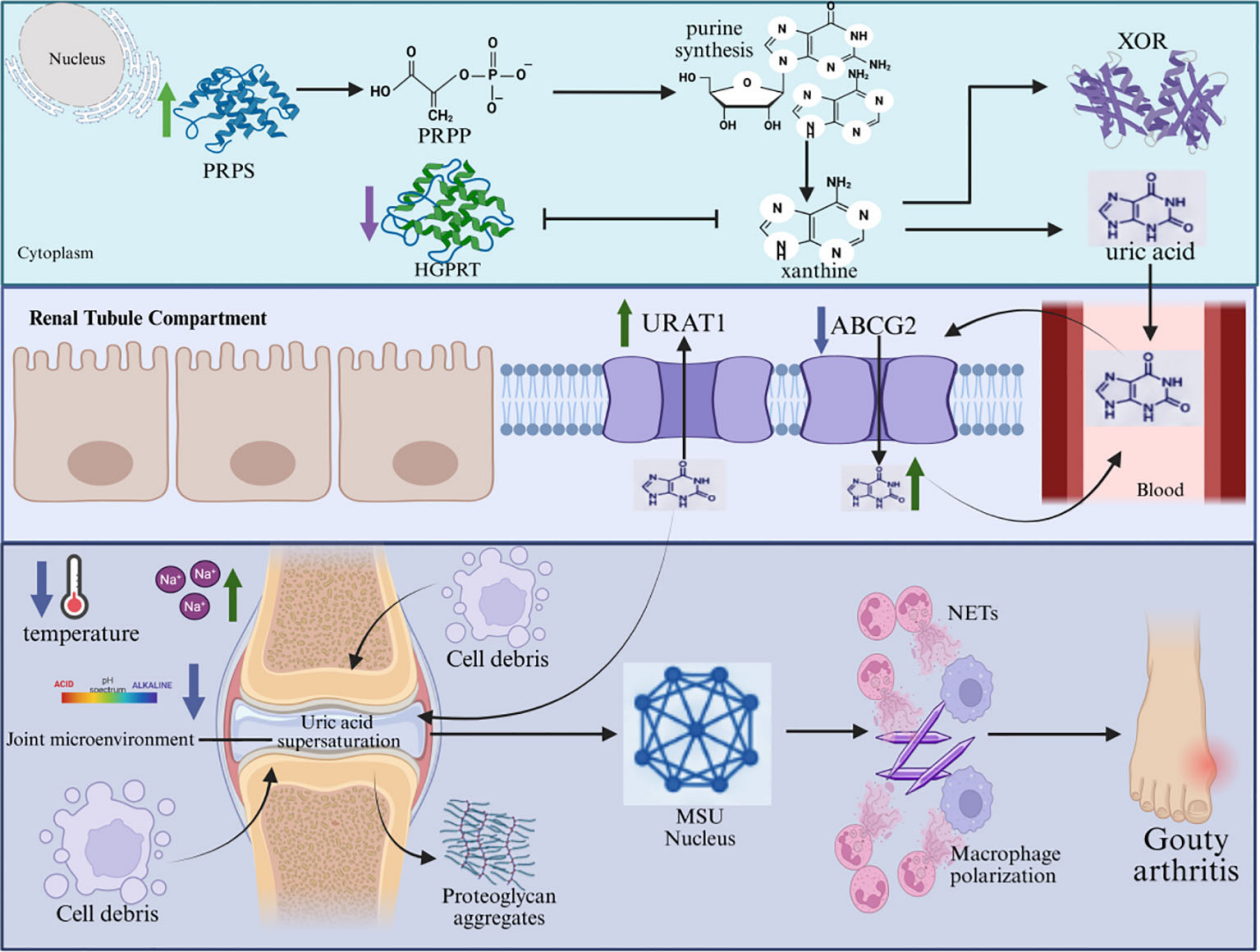
Mechanism of uric acid conversion to MSU crystals. Phosphoribosyl pyrophosphate synthetase (PRPS) enhances uric acid synthesis, whereas hypoxanthine-guanine phosphoribosyltransferase (HGPRT) reduces uric acid production through the purine salvage pathway. Xanthine oxidoreductase (XOR) catalyzes the oxidation of xanthine into uric acid. In renal handling of urate, URAT1 mediates proximal tubular reabsorption, while ABCG2 promotes uric acid excretion. Dysregulation of these metabolic and transport processes elevates serum uric acid levels, resulting in hyperuricemia. Under hyperuricemic conditions, changes in the local joint microenvironment including reduced pH, lower temperature, and increased sodium ion concentration together with cellular debris and proteoglycan aggregates released from damaged cartilage, promote the transition of uric acid into a supersaturated state and initiate MSU nucleation. These nuclei further aggregate and expand, supported by structures such as NETs, ultimately forming MSU crystal deposits that elicit gout-associated inflammatory responses.

Serum uric acid concentrations above 6.8 mg/dL exceed physiological solubility and enter a supersaturated state (17). Once hyperuricemia develops, uric acid becomes supersaturated in peripheral sites such as joint synovial fluid and subcutaneous tissues, enabling the precipitation of MSU crystals. This crystallization process is strongly influenced by the surrounding microenvironment (18). Experimental studies have demonstrated that under uric acid supersaturation, both acidic conditions (pH < 6) and lower temperatures near 35 °C markedly enhance MSU crystal formation (19). Mechanical trauma or physical stress can also promote MSU crystallization by inducing tissue injury and low-grade inflammation. These changes increase vascular permeability, allowing plasma uric acid, proteins, and ions to leak into interstitial spaces, thereby raising local uric acid concentrations and promoting supersaturation (20). Similarly, ischemia or trauma-induced lactic acid accumulation generates an acidic environment that facilitates MSU crystal formation (21). Elevated sodium ion concentrations decrease uric acid solubility and directly favor MSU crystal nucleation and growth (22). In addition, macromolecules such as proteoglycans, which are abundant in synovial fluid, can reduce the apparent solubility of uric acid through “repulsion effects” or specific physicochemical interactions, further promoting crystallization from supersaturated solutions (23). These local microenvironmental shifts explain the predilection of acute gout flares for areas such as the first metatarsophalangeal joint and why surgical interventions or strenuous exercise may precipitate attack (24). Furthermore, another study confirmed that uric acid supersaturation independently predicts cyst expansion in autosomal dominant polycystic kidney disease, highlighting the broader pathological importance of controlling supersaturation in preventing crystal deposition disorders (25). In summary, hyperuricemia provides the systemic foundation for tophus formation, while alterations within the local microenvironment and various external triggers act as the critical determinants of MSU crystal deposition (**Figure 1**).

### 2.2 Nucleation factors promoting sodium urate crystallization

The initiation of MSU crystal formation depends on nucleation, a process in which solute molecules aggregate to form the earliest crystal nuclei within a supersaturated environment. In tophus formation, heterogeneous nucleation is the predominant mechanism, whereby MSU crystals preferentially form on pre-existing solid surfaces, or “nucleation seeds,” rather than spontaneously in a homogeneous solution. These nucleation substrates are commonly associated with joint microinjury or chronic inflammation, which helps explain the predilection of gouty arthritis for weight-bearing or structurally vulnerable joints (17). Several categories of nucleation “seeds” have now been identified. Collagen fibers and cellular debris released from damaged articular cartilage provide surfaces with distinct energy and charge characteristics, effectively lowering the nucleation energy barrier and facilitating the assembly of stable MSU nuclei (26). Microcrystals of hydroxyapatite, as well as pre-existing urate microcrystals, can also serve as direct nucleation sites. Their surface properties enable the adsorption of urate ions, thereby enhancing local supersaturation and accelerating the deposition of larger MSU crystals (27, 28). Components of the extracellular matrix exert a dual influence on the nucleation process. Under pathological conditions such as cartilage degeneration or joint inflammation, degradation of proteoglycans diminishes or removes their natural inhibitory effects on MSU crystallization. In contrast, certain matrix fragments, including chondroitin sulfate oligosaccharides, may shift from being inhibitors to becoming active promoters of nucleation by altering local physicochemical conditions and providing additional binding substrates (29). Low-density lipoproteins have also been shown to function as heterogeneous nucleation substrates, facilitating the deposition of urate under supersaturated conditions (30). Extracellular vesicles, including exosomes and microvesicles, contribute to nucleation regulation through their extensive surface area, diverse molecular binding interfaces, and nucleic acid content, all of which support the assembly of early MSU crystal structures (31). Moreover, positively charged nuclear components released during the formation of NETs, such as DNA and histones can interact electrostatically with uric acid molecules or nascent crystal nuclei. These interactions reduce the energetic threshold required for nucleation, thereby enhancing crystal formation, aggregation, and growth, and establishing a mechanistic link between innate immune activity and urate crystal deposition (17). In summary, MSU crystal nucleation is driven primarily by heterogeneous nucleation. A variety of nucleation-promoting factors, including debris from damaged cartilage, microcrystalline particles, extracellular matrix components altered by pathological conditions, low-density lipoproteins, extracellular vesicles, and materials associated with NETs, work together to reduce nucleation energy barriers and provide surfaces that facilitate crystallization. These factors collectively promote the initiation of MSU crystal formation and contribute to the development of tophi.

## 3 Crystal formation and growth stage: nucleation, growth, and aggregation of sodium urate crystals

### 3.1 Nucleation kinetics and crystal morphology of sodium urate crystals

MSU crystal formation begins with nucleation within supersaturated solutions and is influenced by both classical and non-classical nucleation theories. In classical nucleation, urate ions must overcome an activation energy barrier before they can aggregate into stable initial nuclei (26). The classical nucleation theory, which centers on changes in Gibbs free energy, posits that MSU nucleation is a one-step process involving random collisions and spontaneous assembly of urate anions and sodium ions, with the core mechanism being the formation of crystal nuclei that reach a critical size (32). This theory assumes that crystal nuclei are uniform, dense spherical structures with constant interfacial energy. The nucleation rate is primarily regulated by the solution’s supersaturation and the solid-liquid interfacial energy. Under high supersaturation conditions, homogeneous nucleation predominates, whereas in physiological environments, nucleation is often mediated by heterogeneous nucleation at interfaces with collagen, proteins, and other substances, thereby lowering the nucleation energy barrier (33). Studies have shown that arginine-rich peptides (APs), such as protamine (PRTM), markedly prolong the induction period of MSU nucleation. The guanidino groups of these peptides interact with the surface of amorphous sodium urate (ASU) through hydrogen bonding and electrostatic forces, which stabilizes ASU in a metastable state, raises the nucleation energy barrier, and consequently inhibits nucleation (34). The synergistic interaction between Cu^2+^ and APs further stabilizes ASU, extending nucleation induction time to approximately 48 hours and markedly reducing nucleation rates (35). However, the classical nucleation theory overlooks the existence of metastable intermediate phases and is unable to explain phenomena such as the rapid nucleation of MSU crystals and the formation of amorphous precursors at low supersaturation levels, thus exhibiting certain limitations (36). Non-classical nucleation theories challenge the traditional understanding of single-step nucleation by emphasizing that MSU nucleation first involves the formation of disordered metastable precursors—including amorphous sodium urate clusters and oligomers—which then gradually transform into critical nuclei with stable crystal structures through structural rearrangement, dehydration, or oriented aggregation (37). In non-classical nucleation pathways, molecular regulators such as PRTM stabilize amorphous precursor intermediates and impede their transformation into crystalline phases. These findings highlight key kinetic mechanisms by which biomolecules influence pathological biomineralization processes (38–40). The characteristic needle-like or rod-shaped morphology of MSU crystals arises from anisotropic growth, in which different crystal faces of monosodium urate monohydrate (MSUM) expand at variable rates (34). APs such as PRTM can selectively bind to certain MSUM crystal planes, block growth sites, alter interfacial energy, and reduce the aspect ratio of the crystals, thereby modulating their final morphology (34). DNA molecules can also bind non-selectively to MSU crystal surfaces, inhibit needle-like elongation, and induce a more granular appearance (41). Crystal morphology is closely linked to inflammatory responses. Needle-shaped crystals have a larger surface area relative to volume, making them more easily recognized and engulfed by macrophages. This process activates the NLRP3 inflammasome and stimulates the release of pro-inflammatory cytokines such as interleukin 1 beta (IL-1β) (42). In summary, MSU nucleation kinetics are shaped by both classical and non-classical nucleation mechanisms. Arginine-rich peptides, copper ions, and DNA influence nucleation behavior and crystal morphology through distinct molecular interactions, and the resulting crystal structure plays a critical role in determining the intensity of the inflammatory response.

### 3.2 Crystal growth, aggregation, and stability

The growth, aggregation, and stability of MSU crystals are controlled by both local microenvironmental conditions and specific biomolecules, forming key pathological processes that drive gout and tophus development. Once nucleation has occurred in supersaturated biological fluids, MSU crystals grow primarily through surface-mediated reactions or diffusion-dependent mechanisms. Their typical needle-like morphology is particularly important in eliciting strong inflammatory responses, because the elongated shape increases surface area and promotes penetration of cellular membranes, which subsequently activates intracellular inflammatory pathways (43, 44). A range of biomacromolecules and small molecules can modulate crystal growth and morphology. DNA can adsorb nonspecifically to crystal surfaces and inhibit further elongation, thereby transforming needle-like structures into granular forms (45). Theophylline, which has a structural resemblance to uric acid, can incorporate into the crystal lattice and slow crystal growth by altering structural integrity (46). MSU crystals preferentially accumulate in regions characterized by slow blood flow, reduced temperature, or repeated mechanical stress, such as the first metatarsophalangeal joint and the auricle (47). Low temperatures decrease uric acid solubility, while hypoxic and acidic conditions encourage crystal precipitation (11). Crystal deposition often follows the orientation of collagen fibers in the extracellular matrix, and macrophage subpopulations within developing tophi contribute to fibrotic tissue remodeling surrounding the deposited crystals (48). Crystal aggregation is a crucial step in tophus formation and is largely driven by van der Waals forces and electrostatic interactions (49). Aggregated crystals tend to persist within tissues and provoke sustained inflammatory responses, with larger aggregates correlating with more severe disease manifestations (50). Certain inhibitors, such as theobromine, effectively limit MSU crystal aggregation (51, 52). Additionally, serum proteins can form a “protein crown” on the crystal surface, while adsorbed molecules including DNA and melamine alter crystal surface characteristics. These modifications influence crystal stability, immune recognition, and cellular interactions by altering surface properties (53). This, in turn, influences crystal clearance and the formation and dissolution kinetics of tophi (10). In summary, MSU crystal growth, aggregation, and stability are shaped by a combination of microenvironmental factors and regulatory biomolecules or small molecule modulators. The resulting crystal morphology, deposition patterns, aggregation behavior, and surface modifications directly contribute to the initiation, persistence, and progression of gout and tophus formation.

## 4 Acute inflammation trigger phase: the encounter between crystals and the innate immune system

### 4.1 Crystal recognition and endocytosis by pattern recognition receptors

MSU crystals act as the primary triggers of gouty inflammation and are detected and internalized by innate immune cells through pattern recognition receptors (PRRs). This recognition is essential for initiating downstream inflammatory pathways. Macrophages and other immune cells identify MSU crystals, along with their associated protein coatings, through both surface and intracellular PRRs. The scavenger receptor MARCO can directly bind unmodified MSU crystals and facilitate their uptake (54). CD44, a cell surface adhesion molecule, also regulates macrophage phagocytosis. Cleavage of its extracellular domain enhances the initial attachment of MSU crystals and promotes their internalization (55). Intracellular NOD-like receptors (NLRs), as core cytoplasmic PRRs, respond to danger-associated molecular patterns like MSU crystals. Among these, NLRP3 plays a central role and is more critical for MSU sensing than classical Toll-like receptors. NLRP3 detects intracellular disturbances and assembles the inflammasome complex in response to crystal-mediated stress signals (56, 57). After phagocytosis of MSU crystals, destabilization or rupture of the phagosomal membrane becomes a pivotal event for NLRP3 activation. This disruption triggers ionic imbalances, most notably potassium efflux, along with the generation of reactive oxygen species (ROS) and lysosomal injury. These events enhance phagosomal permeabilization, allowing crystal-related signals to be detected by NLRP3, which subsequently activates caspase-1 and promotes the maturation and secretion of IL-1β (58). The physicochemical characteristics of MSU crystals, including their size, morphology, surface chemistry, and the nature of surface-bound proteins, influence receptor specificity and the efficiency of phagocytosis. In addition, the intrinsic state of the host cell determines how it processes the crystals. Together, these factors shape the mechanisms underlying immune recognition and inflammatory activation in gout (59, 60).

### 4.2 Activation of the NLRP3 inflammasome and maturation/release of IL-1β

MSU crystals act as the central initiators of tophus formation by activating the NLRP3 inflammasome and inducing the maturation and release of IL-1β through a tightly regulated two-step signaling process. In the first step, pattern recognition receptors such as Toll-like receptors detect MSU crystals and activate the nuclear factor kappa B (NF-κB) signaling pathway (61). This activation increases the transcription of NLRP3 and pro-IL-1β, establishing the necessary protein pool for subsequent inflammasome assembly (62). Additional pathways, including TLR-mediated signaling, MAPK pathways, and mTOR signaling, further modulate this priming stage (63, 64). In the second step, following phagocytosis of MSU crystals by macrophages, a series of intracellular stress signals are generated. These include potassium efflux, lysosomal rupture accompanied by the release of cathepsin B, and mitochondrial dysfunction associated with increased production of ROS (65–67). Soluble uric acid can also modulate ROS via UCP2 (68). Mitochondrial DNA oxidation, lactate-induced modification of NLRP3, and the interaction of NLRP3 with the adaptor protein NEK7 further contribute to inflammasome activation by promoting NLRP3 conformational changes and oligomerization (69–71). Once oligomerized, NLRP3 recruits the adaptor protein ASC through pyrin domain interactions. ASC then recruits pro-caspase-1 through its caspase recruitment domain, forming the fully assembled NLRP3 inflammasome complex (72). This complex triggers the self-cleavage of caspase-1 into its active form (73). Active caspase-1 cleaves pro-IL-1β and pro-IL-18 into their mature forms, which are released through membrane pores formed by gasdermin D. This process also leads to pyroptotic cell death, further amplifying the inflammatory response (74, 75). Mature IL-1β functions as a central mediator of gouty inflammation. Through autocrine and paracrine binding to IL-1 receptors, it intensifies inflammatory signaling and promotes the expression of cytokines such as TNF-α and IL-6, as well as chemokines including CXCL1 and IL-8. Among these mediators, CXCL1 plays a key role by recruiting neutrophils to the inflamed joint, where they further amplify inflammation through the release of NETs and related mechanisms, ultimately driving acute gouty arthritis (76, 77). The clinical effectiveness of IL-1β-targeting agents, such as anakinra, supports the central importance of this pathway in disease pathogenesis (78). In GA, in addition to the classical NLRP3 inflammasome pathway, the AIM2 inflammasome and IL-18 also play important roles. As a cytoplasmic DNA sensor, AIM2 can be activated by dsDNA released from NETs induced by MSU crystals; upon binding to ASC, it recruits and activates Caspase-1, forming a functional inflammasome. Activated caspase-1 cleaves GSDMD to induce pyroptosis, while simultaneously catalyzing the maturation and secretion of IL-1β and IL-18 precursors (79). Upon binding to its receptor, mature IL-18 activates the NF-κB signaling pathway, promoting the secretion of pro-inflammatory factors such as TNF-α and IL-6, thereby amplifying the local inflammatory response in the joint and exacerbating synovial damage (80). Clinical studies have confirmed that the expression of AIM2, caspase-1, and GSDMD proteins is significantly elevated in peripheral blood mononuclear cells of patients with acute gouty arthritis, and these levels correlate positively with inflammatory markers, suggesting that AIM2-mediated pyroptosis is a critical component of acute gout attacks (81). In the chronic tophaceous environment, persistent exposure to MSU crystals repeatedly activates the NLRP3 inflammasome and sustains a state of chronic low-grade inflammation (82). Crosstalk between classical and non-classical inflammasome pathways, partly mediated by caspase-8, may further maintain IL-1β production and contribute to progressive tophus growth. Consequently, NLRP3 small-molecule inhibitors have emerged as a potential therapeutic strategy (83, 84). In summary, MSU crystals activate the NLRP3 inflammasome through a well-defined two-step signaling process that leads to caspase-1 activation and the cleavage of pro-IL-1β into its mature form. This pathway also contributes to pyroptosis, collectively driving both the acute inflammatory episodes characteristic of gout and the chronic inflammatory processes that underpin tophus formation and progression.

## 5 Inflammation amplification and cell recruitment phase: the dominant role of neutrophils

### 5.1 Neutrophil infiltration and activation

During the early phase of tophus development, neutrophil recruitment and activation form the central processes that initiate and amplify the inflammatory response. These events are orchestrated by an intricate signaling network involving chemokines, inflammatory mediators, and complement components. IL-1β, a principal initiator of gouty inflammation, works together with multiple chemokines to guide neutrophils toward sites of MSU crystal deposition. CXCL1 and CXCL2, the major ligands for the CXCR2 receptor, are produced following NLRP3 activation and promote neutrophil infiltration through the CXCL1/2–CXCR2 axis (85). CXCL5 enhances this process by activating CXCR2 on nociceptive neurons, thereby intensifying neutrophil chemotaxis, joint inflammation, and pain (86). The complement fragment C5a binds to its receptor C5aR1 on target cells and further amplifies inflammatory signaling while driving additional neutrophil recruitment (87). Together, these mediators facilitate neutrophil extravasation and accumulation at the site of inflammation. Once neutrophils reach the deposition area, the large size of MSU crystals leads to “frustrated phagocytosis,” where unsuccessful engulfment triggers the release of lysosomal enzymes and reactive oxygen species. MSU crystals have been shown to directly induce ROS production in neutrophils (88), and these ROS contribute significantly to local tissue damage (89). Concurrently, leukotriene B4 (LTB4) also plays a key role by enhancing neutrophil recruitment and stimulating other immune cells, thereby worsening tissue injury (90). In addition, activated neutrophils release inflammatory mediators such as myeloperoxidase (MPO), which promotes synovial cells and macrophages to produce higher levels of IL-1β, CXCL8, and other cytokines (91, 92). This establishes a positive feedback loop that perpetuates inflammation and creates the conditions necessary for tophus formation. In summary, neutrophil infiltration and activation are central drivers of early gouty inflammation. Targeting critical pathways such as CXCR2 signaling and neutrophil-derived inflammatory mediators may offer effective strategies for managing acute gout attacks and preventing progression toward chronic tophaceous disease.

### 5.2 Formation of NETs and their double-edged sword effects

In the development of tophi, NETs exert a critical yet paradoxical influence. Their dysregulated formation and impaired clearance contribute directly to the shift from acute gouty inflammation to chronic granulomatous disease (93–95). NETs are generated through the process of NETosis, and the distinction between these two pathways lies in whether neutrophils undergo cell death following NET formation. The non-NADPH oxidase-dependent pathway (also known as viotic NETosis) does not rely on PAD4 activity. In contrast, apoptotic NETosis requires PAD4 to mediate histone methylation, which leads to chromatin depolymerization. NETs are generated through NETosis, a specialized form of neutrophil death triggered by MSU crystals. This process involves chromatin decondensation, mixing of nuclear and granule components, and eventual rupture of the plasma membrane. The released NET structure consists of a DNA backbone coated with histones, MPO, neutrophil elastase (NE), and related proteins (96). NET formation proceeds through two principal pathways. The NADPH oxidase–dependent pathway requires the production of reactive oxygen species, nuclear translocation of NE and MPO, and histone modifications that promote chromatin disorganization (59). The NADPH-independent pathway depends on peptidylarginine deiminase 4 (PAD4), which catalyzes histone citrullination and drives chromatin relaxation. Actin cytoskeletal remodeling is essential for NET release by supporting NE entry into the nucleus and facilitating DNA extrusion. MSU crystals are potent physiological inducers of NETosis in the context of gout (97–99). The functional consequences of NETs are closely tied to their molecular composition. Granule-derived proteins, including modified histones, MPO, NE, and calmodulin, embedded within the DNA scaffold, can entrap MSU crystals and restrict their spread within the joint. This sequestration helps limit early inflammatory damage and resembles the role of NETs in mineral deposition during kidney stone formation, where they facilitate calcium salt aggregation (100, 101). Despite these protective effects, NETs also contribute to pathological injury. Excessive NET formation or inadequate NET clearance promotes MSU crystal aggregation, creating a structural framework for chronic granuloma formation at the center of tophi (10). At the same time, NET components such as extracellular DNA, citrullinated histones, and MPO act as autoantigens. These molecules can activate antigen-presenting cells, including plasmacytoid dendritic cells, thereby inducing autoantibody production and sustaining chronic inflammation. This mechanism is a major driver of persistent granulomatous tissue formation in tophi (102). NETs can also degrade inflammatory cytokines and contribute to resolution during gout remission, which may help prevent flare recurrence (103). However, when NETs accumulate continuously under pathological conditions, they intensify tissue damage. This detrimental effect parallels the role of NETs in promoting disease progression in conditions such as biliary atresia, malignancy, and atherosclerosis (104–107).

**Figure 2:**
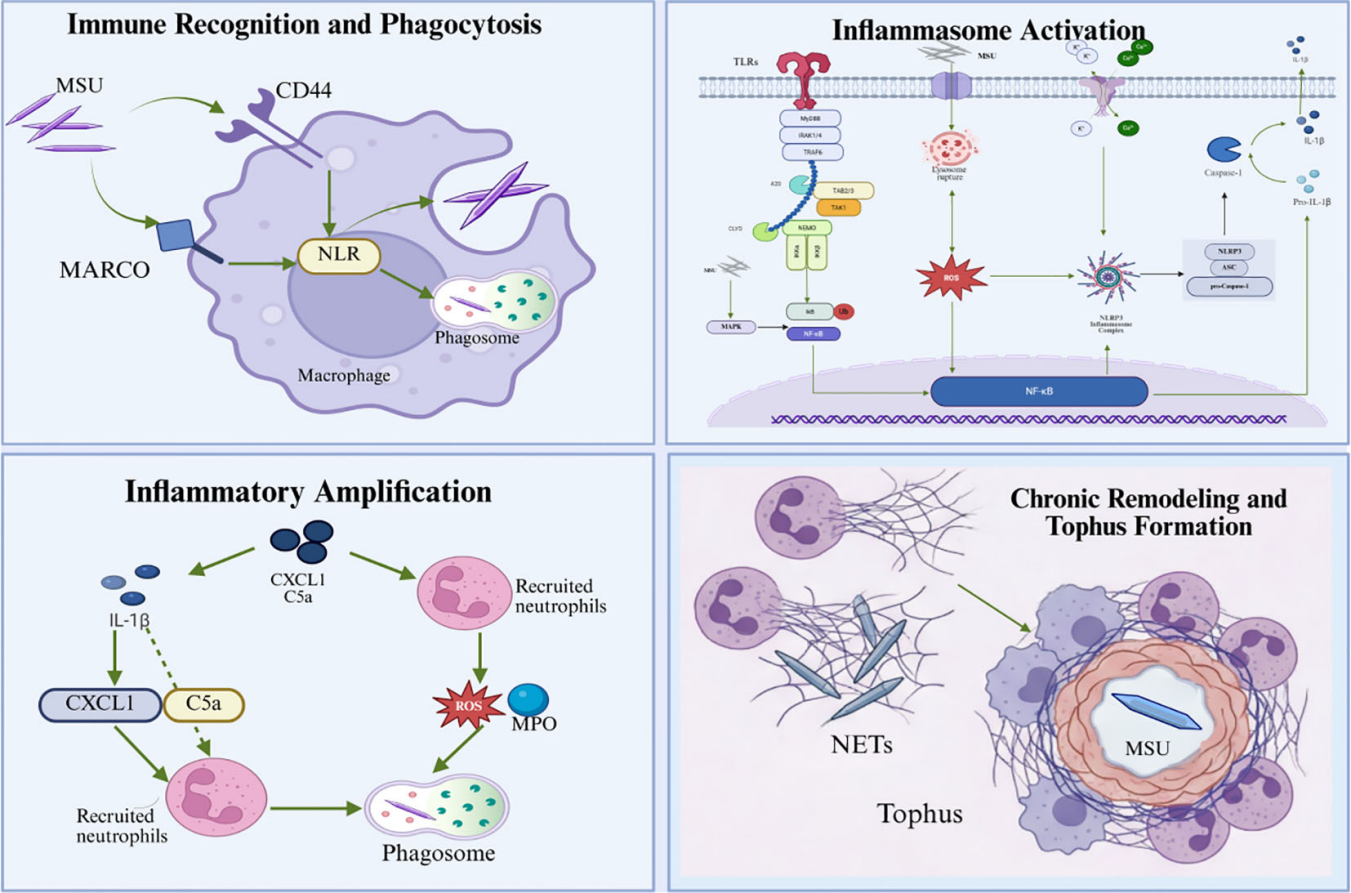
Core pathogenic mechanism of MSU-mediated tophi formation. This figure illustrates the four major stages through which MSU crystals promote tophus development: innate immune recognition, inflammasome activation, inflammatory amplification, and chronic tissue remodeling. Innate immune recognition and endocytosis: Macrophages detect and internalize MSU crystals through surface receptors such as the scavenger receptor MARCO and CD44, as well as intracellular NOD-like receptors. This initial interaction triggers downstream inflammatory signaling. Inflammasome activation: MSU activates NF‑κB via TLR‑MyD88 and MAPK signaling to upregulate NLRP3 and pro‑IL‑1β transcription. Endocytosed MSU triggers lysosomal rupture, ROS accumulation, K^+^ efflux and Ca^2+^ influx, facilitating assembly of NLRP3‑ASC‑pro‑Caspase‑1 inflammasome. Activated caspase‑1 cleaves pro‑IL‑1β to produce secreted mature IL‑1β and initiate inflammation. Inflammatory amplification: Mature IL-1β stimulates the production of chemokines such as CXCL1 and complement component C5a, driving neutrophil recruitment to the site of crystal deposition. Infiltrating neutrophils attempt to phagocytose MSU crystals and release reactive oxygen species, myeloperoxidase, and other inflammatory mediators, creating a self-perpetuating inflammatory loop. Chronic remodeling and tophus formation: NETs encapsulate and promote the aggregation of MSU crystals. Their persistent presence sustains chronic inflammation and facilitates the development of crystal-centered granulomatous tissue, ultimately leading to tophus formation.

The clearance of NETs primarily relies on DNase-mediated extracellular degradation and macrophage-mediated phagocytic degradation. As the major extracellular DNAase in serum and synovial fluid, DNase I hydrolyzes the DNA scaffold of NETs; however, serum DNase I activity is significantly reduced in patients with GA, creating a vicious cycle (108); DNase II acts in concert with macrophages to complete the thorough clearance of NETs (109). Their formation and regulation are governed by the interaction of multiple pathways; the NADPH oxidase/ROS pathway is the core driver of MSU crystal-induced NETosis. MSU crystals promote ROS production by activating relevant receptors and signaling pathways, thereby driving NET release and activating NLRP3 inflammasome formation, creating a pro-inflammatory positive feedback loop (110); inflammasomes and autophagy interact and regulate each other through signaling axes such as mTOR-ATG7-PAD4. The NLRP3 inflammasome accelerates NET release, while autophagy influences NET formation by regulating histone citrullination (111); purinergic signaling and the inflammatory microenvironment further fine-tune NET homeostasis, with pro-inflammatory factors amplifying NETosis, whereas anti-inflammatory factors suppress NET formation and enhance their clearance capacity (112).In summary, NETs participate in early MSU crystal sequestration but also connect acute inflammation with chronic pathological remodeling. Their dual protective and harmful functions make them key regulators of tophus initiation, structural development, and progression (**Figure 2**).

## 6 Inflammation resolution and transformation phase: transition to chronic granuloma

### 6.1 Macrophage phenotypic switch: from M1 to M2

In the late phase of acute gout, changes in the local inflammatory milieu act as the principal drivers of macrophage phenotype transition from the proinflammatory M1 state to the reparative M2 state. As inflammation begins to resolve, concentrations of cytokines such as TNF-α and IL-1β progressively decline, whereas levels of anti-inflammatory and pro-repair mediators including TGF-β and IL-10 increase (113). This evolving cytokine environment induces phenotypic reprogramming in macrophages recruited to sites of MSU deposition. M1 macrophages are characterized by high inducible nitric oxide synthase expression and robust production of proinflammatory cytokines, and they function primarily as mediators of acute inflammation (114). In contrast, M2 macrophages express markers such as arginase-1, CD206, and CD163, and they participate in anti-inflammatory responses and tissue repair (115). Meanwhile, in the progression of gouty arthritis, single-cell transcriptomics can characterize the transcriptional profiles and functional differences between M1 and M2 macrophage subpopulations (116) scRNA-seq reveals that core transcription factors such as NF-κB1 and STAT6, as well as non-coding RNAs including miR-155 and the lncRNA MALAT1, regulate the polarization process; cells participate in inflammatory regulation through ligand-receptor interactions such as CXCL8-CXCR2 and TGF-β-TGFβR (117, 118). Several signaling pathways regulate macrophage polarization. The aryl hydrocarbon receptor promotes M2 polarization and suppresses the M1 phenotype through the AhR–miR-142a–IRF1/HIF-1α regulatory axis (119). Lipoxygenase A4 (LXA4) suppresses M1 polarization by downregulating NF-κB p65 and IRF5 activity through the FPR2-IRF pathway, and induces M2 polarization via the FPR2/IRF4 signaling pathway (113). However, the hyperuricemic environment in gout disrupts these regulatory mechanisms. Soluble uric acid can interact with TLR2 agonists to decrease M2 marker expression and increase proinflammatory gene transcription, shifting macrophages toward an M1-like phenotype (120). However, elevated TGF-β and IL-10 in the acute late phase remain the dominant forces driving M2 conversion and initiating inflammation control and repair (121). Once macrophages transition to the M2 phenotype, their primary function is to phagocytose and clear deposited MSU crystals. Failure to achieve effective clearance triggers programs associated with fibrotic encapsulation. A specialized macrophage subpopulation located at the periphery of tophi, marked by SPP1, MMP9, and CHI3L1 expression, secretes MMP9 and osteopontin to remodel the extracellular matrix. These macrophages adopt a fibroblast-like phenotype and participate in fibrotic tissue formation (122). Concurrently, TGF-β produced by M2 macrophages stimulates fibroblast proliferation and differentiation, promoting collagen deposition that encapsulates MSU crystals and contributes to tophus development (121). Additionally, M2 macrophages M2 also participate in efferocytosis by clearing apoptotic neutrophils through the MerTK receptor. During this process, they secrete IL-10 and TGF-β, reinforcing an anti-inflammatory feedback loop (121). When these regulatory mechanisms fail, apoptotic cell debris accumulates and persistently activates innate immune pathways, which can destabilize or even reverse the M2 anti-inflammatory phenotype (123). This dysfunction sustains chronic inflammation and drives the progression of gout toward persistent tophus formation. In summary, the balance between M1 and M2 macrophage phenotypes is a critical determinant of inflammatory resolution in gout. Dysregulation of the M1-to-M2 transition represents a central mechanism linking acute gouty inflammation with chronic tissue remodeling and tophus development.

### 6.2 Activation of fibroblasts and extracellular matrix remodeling

During tophus development, fibroblast activation is a central event that initiates extracellular matrix remodeling. Together, fibroblast activation and ECM restructuring form the core pathological foundation that drives the transition of MSU deposit sites from acute inflammatory lesions to chronic, structurally organized tophi. MSU crystals, infiltrating macrophages, and macrophage-derived cytokines create a specialized microenvironment that induces the conversion of resting fibroblasts into activated myofibroblasts. This transformation is primarily driven by the TGF-β1 signaling pathway (124, 125). TGF-β1, produced in part by M2 macrophages, acts in a paracrine manner to amplify fibroblast activation, leading to increased proliferation and robust synthesis of ECM components, especially type I and type III collagen (126). Multiple regulatory mechanisms govern fibroblast activation and ECM deposition. AMP-activated protein kinase suppresses fibroblast activation and abnormal ECM accumulation by inhibiting Smad3 signaling (127). As ECM stiffness increases, mechanotransduction through integrins further enhances fibroblast activation and phenotypic transformation, acting synergistically with TGF-β1 (128, 129). Collagen fibers produced by early activated fibroblasts form a loose matrix network that establishes the initial granuloma scaffold. This structure both physically isolates MSU crystals from surrounding tissues and provides anchoring sites for subsequent cell migration and denser collagen deposition (130). The direction of ECM remodeling depends on the dynamic balance between matrix metalloproteinases (MMPs) and tissue inhibitors (TIMPs). In the early stages, MMPs such as MMP-1, MMP-3, and MMP-13 are transiently upregulated to clear cellular debris and remodel damaged tissue (131). As the lesion matures, TGF-β1 increases the expression of tissue inhibitors of metalloproteinases, reducing ECM degradation and favoring net collagen accumulation, which progressively consolidates the initially loose collagen network (132). Furthermore, MSU crystals within tophi continuously induce local chronic inflammation, leading to extracellular matrix remodeling and increased tissue stiffness. Through the integrin-FAK-YAP/TAZ signaling pathway, they mediate the nuclear translocation of YAP/TAZ, which, upon binding to the TEAD transcription factor, upregulates fibrosis-related target genes such as Col1A1, α-SMA, and TGF-β1, thereby inducing the transformation of fibroblasts into myofibroblasts (133). Concurrently, MSU promotes the secretion of Wnt ligands by macrophages and fibroblasts, inhibits β-catenin degradation, and facilitates its nuclear accumulation, thereby activating downstream fibrosis-related transcriptional programs (134). YAP/TAZ and the Wnt/β-catenin pathway exhibit bidirectional positive regulation, synergistically amplifying the fibrotic response; TGF-β simultaneously activates Smad and YAP/TAZ signaling and induces Wnt ligand expression, achieving cross-coupling between mechanical stimulation, inflammatory responses, and fibrotic pathways, which collectively drive the progression of pathological fibrosis in tophi (135). In the chronic phase, persistent activation signals from factors including TGF-β and PDGF lead to sustained fibroblast activity and continuous ECM production (136–138). The resulting dense fibrous capsule partially mitigates acute inflammation by isolating MSU crystals but also hinders the penetration of therapeutic agents (139). Over time, irregular collagen organization and aberrant cross-linking increase tissue stiffness and perpetuate fibroblast activation through mechanical feedback mechanisms, contributing to progressive joint impairment (140, 141). In addition, excessive MMP activity at the periphery of tophi promotes local joint destruction, whereas the dominance of TIMPs within the tophus core stabilizes the fibrotic structure. This spatially and temporally coordinated pattern of ECM turnover creates a pathological network that sustains tophus growth and chronicity (142, 143). Together, these processes illustrate that fibroblast activation and ECM remodeling are not merely secondary consequences of inflammation but are fundamental drivers of tophus maturation, structural stability, and long-term disease progression, highlighting them as critical therapeutic targets in chronic gout.

### 6.3 Coagulation dysfunction (hypercoagulable state) promotes tophi formation

Coagulation dysfunction, manifesting as a hypercoagulable state, acts as a major amplifying factor in the formation and progression of tophi. Its central mechanism involves a coordinated network that activates the coagulation–inflammation axis, alters the local tissue microenvironment, promotes crystal aggregation, and accelerates fibrotic remodeling, ultimately driving the chronic evolution of gout (2, 144). In hypercoagulable conditions, elevated thrombin generation not only catalyzes fibrin formation (145, 146) but also functions as a pro-inflammatory mediator. Through activation of protease-activated receptors, thrombin stimulates endothelial cells and monocyte/macrophage populations to release cytokines such as IL-1β and IL-6, thereby recruiting additional inflammatory cells and establishing a self-reinforcing “coagulation–inflammation” positive feedback loop. At the same time, thrombin promotes M1 macrophage polarization, enhancing their capacity to phagocytose MSU crystals and intensifying inflammatory mediator release, which contributes to the early inflammatory framework necessary for tophus formation. Fibrin deposition and NETs enhancement further impede crystal clearance and promote granuloma formation (95, 147, 148). The hypercoagulable state also acts synergistically with MSU crystals to injure the vascular endothelium, resulting in microcirculatory dysfunction. This leads to local ischemia, hypoxia, reduced pH, and decreased temperature, all of which lower uric acid solubility and impair urate excretion. This creates a vicious cycle of “local hyperuricemia-crystal deposition-microcirculatory deterioration,” accelerating crystal precipitation (149–151). At the same time, activated platelets release mediators such as P-selectin and TGF-β, which intensify local inflammation, promote microthrombosis, and stimulate fibroblast proliferation. Fibrinogen and coagulation factor XII can directly bind to MSU crystals, enhancing their aggregation and fusion and thereby creating a microenvironment favorable for tophus enlargement (152, 153). Clinical evidence indicates that patients with gout commonly display hypercoagulable features, including platelet activation, elevated coagulation factors, increased fibrinogen, and higher D-dimer levels. Individuals who also present with deep vein thrombosis or thrombotic microangiopathy tend to have a greater incidence of tophi and more rapid tophus progression (154). In summary, coagulation abnormalities do not initiate tophus formation but function as powerful amplifiers of chronic gout pathology. Thus, by activating the coagulation–inflammation axis, worsening the local microenvironment, promoting urate crystal aggregation, and accelerating tissue fibrosis, hypercoagulability contributes significantly to tophus development and persistence. Future research may benefit from exploring strategies that target or correct hypercoagulable states as a means to slow or prevent tophus formation.

## 7 Granuloma formation stage: construction of chronic inflammatory tissue

### 7.1 Formation and function of multinucleated giant cells

Multinucleated giant cells (MGCs) are specialized cell types that arise through the fusion of macrophages under defined pathological conditions. They mainly include foreign body giant cells (FBGCs) and Langerhans giant cells (LGCs) (155). Their formation involves a coordinated sequence of intercellular recognition, adhesion, and membrane fusion events that depend on the regulated expression of specific surface molecules and signaling pathways. Studies have shown that during IL-4–mediated induction of MGCs, the C-type lectin family member CD301 (CLEC10A) is markedly upregulated and promotes intercellular adhesion and fusion (156). The transmembrane receptor TREM2, acting through its adaptor protein DAP12, is also essential for promoting macrophage fusion and multinucleation, particularly in lipid-rich environments such as obesity-associated adipose tissue, where TREM2 functions as a marker of lipid-associated macrophages (157). Common mononuclear progenitor cells (CMPCs) with high lipid and cholesterol storage capacity have been identified as potential precursors of MGCs and can be recruited into granulomas under pathological conditions, including infection (158). The fusion process, represents an advanced maturation step for macrophages, allowing them to acquire the unique functional and transcriptional identity that characterizes MGCs (159). In tophaceous granulomas, MGCs, particularly foreign body giant cells, position themselves around aggregated MSU crystals. In addition, by physically encapsulating urate deposits that cannot be cleared by individual macrophages, they contribute directly to granuloma formation and maintenance (160). MGCs perform analogous functions in other disease settings. In adipose tissue of obese individuals, they clear necrotic adipocytes, whereas in experimental models of intracerebral hemorrhage, they phagocytose large numbers of red blood cells and express genes associated with hemoglobin degradation (161, 162). MGCs also act as antigen-presenting cells and as sources of cytokines that regulate chronic inflammation. Langerhans giant cells express high levels of B7-H3, which enhances T-cell activation (142). IL-15 drives LGC formation via CD40L-CD40 interactions and IFN-γ release (163). while IL-4-induced MGCs lose partial cytokine secretion capacity to limit excessive inflammation (164). In the chronic inflammatory environment of tophi, MGCs play essential roles in maintaining granuloma stability and sustaining immune activation. They present crystal-associated antigens, produce inflammatory mediators, and interact with surrounding immune cells, thereby contributing to the persistence and progression of chronic gout (165). In conclusion, MGCs are not merely bystanders within tophi but are active participants in granuloma organization, antigen presentation, and chronic inflammatory regulation. Their formation and function represent key cellular mechanisms that support the long-term stability of tophi and promote the chronicity of gout.

### 7.2 Complex cellular infiltration and angiogenesis

Tophi, the defining pathological structures of chronic gout, arise through the coordinated actions of complex immune cell infiltration, persistent inflammation, and pathological angiogenesis rather than MSU crystal deposition alone. Cellular infiltration is a central feature of the chronic inflammatory microenvironment within tophi. In addition to macrophages and fibroblasts, both adaptive and innate immune cells contribute significantly to this process. Prominent infiltration of Th1 and Th17 cells has been documented in tophaceous tissue (166). The IFN-γ secreted by further enhances macrophage activation and strengthens their proinflammatory phenotype, while IL-17 secreted by Th17 cells exerts strong proinflammatory and profibrotic effects by stimulating fibroblast proliferation and promoting collagen deposition (166). Mast cells further amplify local inflammation by releasing histamine, proteases, and cytokines. These mediators not only worsen inflammatory injury but also facilitate the structural maturation and enlargement of tophi by modulating vascular permeability and influencing fibroblast function, thereby contributing to a complex immune–inflammatory network. Pathological angiogenesis, which closely accompanies immune cell infiltration, is another essential driver of tophus progression. This abnormal vascular formation is sustained by proangiogenic factors such as VEGF and FGF, which are produced by infiltrating inflammatory cells, activated fibroblasts, and endothelial cells within the lesion (167). The dense cellular composition of tophi, together with impaired vascular function, frequently generates a distinct hypoxic microenvironment. Under these conditions, the stability of HIF-1α is markedly increased because its degradation is inhibited. Stabilized HIF-1α enhances the expression of VEGF, promoting the formation of structurally disorganized and highly permeable neovessels that resemble the abnormal vasculature seen in chronic non-healing wounds (168). These newly formed vessels play two critical roles. They provide oxygen and nutrients to infiltrating immune cells and activated fibroblasts, thereby directly supporting tophus growth (168). This mechanism parallels inflammation-driven angiogenesis in colon cancer, where macrophage-derived CXCL12 promotes vascular growth (169). At the same time, these vessels function as conduits that facilitate continual infiltration of immune cells and inflammatory mediators, creating a self-reinforcing cycle of inflammation and angiogenesis. This relationship reflects the reciprocal regulation between inflammatory pathways and angiogenic signals observed in rheumatic diseases (170). The combined actions of immune cell infiltration, pathological angiogenesis, and extracellular matrix remodeling shape the characteristic multi-zonal architecture of tophi and define their progressive nature. In summary, the intricate interplay between inflammatory cell accumulation and aberrant angiogenesis sustains the chronic inflammatory environment within tophi. Therefore, therapeutic strategies that target upstream mediators such as VEGF or key inflammatory signaling pathways may offer promising avenues for controlling tophus progression (171, 172).

## 8 Maturation and stabilization stage: fibrous encapsulation and tissue destruction

### 8.1 Formation and histological characteristics of the dense fibrous capsule

**Figure 3:**
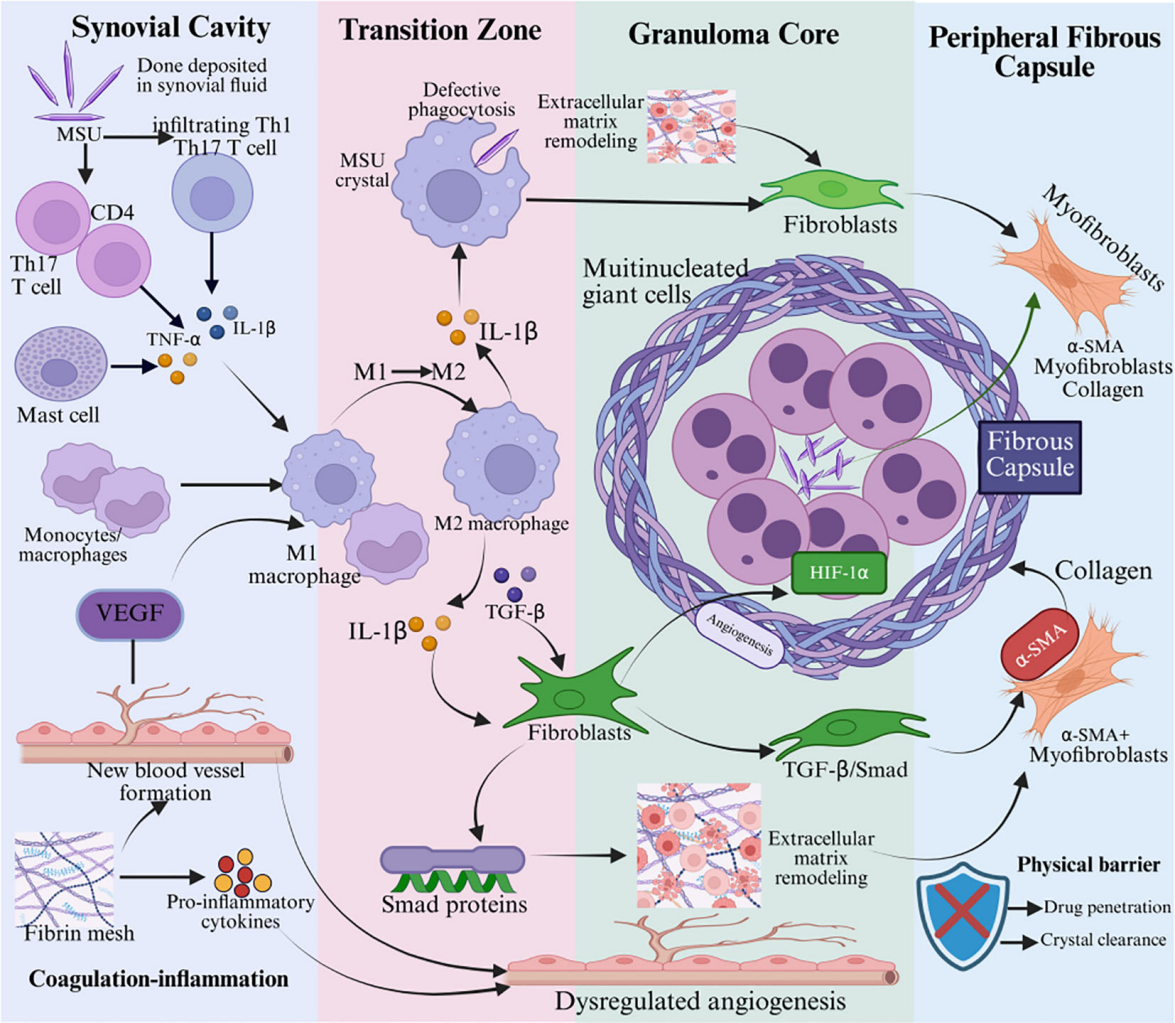
Mechanism of chronic granuloma formation in tophi. This figure illustrates the pathogenic process through which tophi develop, driven by the combined effects of MSU crystal–induced chronic inflammation and dysregulated tissue repair. The central mechanism involves the transition from acute inflammatory responses to chronic granulomatous organization, ultimately resulting in formation of a dense fibrotic capsule. The major pathological zones and associated molecular and cellular events are summarized below. Synovial cavity zone: Deposition of MSU crystals activates innate and adaptive immune pathways, leading to infiltration of Th1 and Th17 cells as well as mast cells. These cells release proinflammatory mediators, including IL-1β and TNF-α, which promote M1 macrophage polarization. At the same time, increased VEGF expression stimulates angiogenesis, and activation of the coagulation–inflammation axis further amplifies local inflammation. Transition Zone: Continued inflammatory signaling induces the shift from M1 to M2 macrophage phenotypes. M2 macrophages display reduced phagocytic capacity toward MSU crystals, release IL-1β, and secrete TGF-β, thereby initiating fibrotic signaling pathways. Granuloma Core Zone: At the center of the lesion, MSU crystals become enclosed by multinucleated giant cells, forming the structural core of the granuloma. Elevated HIF-1α expression enhances angiogenesis and supports sustained inflammatory activity in this hypoxic microenvironment. Peripheral Zone: TGF-β drives fibroblast transdifferentiation into α-SMA–positive myofibroblasts through Smad-dependent pathways. These activated fibroblasts produce large amounts of collagen, generating a dense fibrous capsule that limits drug penetration and reduces the efficiency of crystal clearance. Overall, the interplay of immune cell polarization, extracellular matrix remodeling, activation of the coagulation–inflammation axis, and aberrant angiogenesis shapes the progression of tophi. This coordinated process forms the fundamental pathological basis for chronic gout and contributes to progressive joint damage.

Tophus formation is a pathological process that integrates persistent inflammation caused by urate crystal deposition with dysregulated tissue repair. The dense fibrous capsule that characterizes mature tophi forms the structural core of this process and originates from sustained fibroblast activation and phenotypic transformation driven by the chronic inflammatory microenvironment (173). This progression reflects a continuum from acute inflammatory responses to chronic granuloma organization, ultimately culminating in the development of a tightly layered fibrous capsule surrounding the lesion (**Figure 3**). During the chronic stage, tophi continually release cytokines such as TGF-β into the local environment. Activation of the TGF-β–Smad signaling pathway promotes the differentiation of fibroblasts into myofibroblasts. These activated myofibroblasts exhibit strong contractile function and robust extracellular matrix synthesis, producing large quantities of type I and type III collagen. Under the influence of cross-linking enzymes, these collagen fibers become densely cross-linked, forming a compact fibrotic capsule. This encapsulation represents a protective attempt by the host to isolate urate crystals that cannot be effectively cleared (174). Mature tophus capsules show distinct histological characteristics, consisting largely of dense fibrous connective tissue with sparse cellularity and limited vascularization (46). This structure provides mechanical stability and acts as a physical barrier that restricts urate crystal diffusion and limits surrounding tissue erosion. However, this protective architecture has detrimental consequences. The densely packed collagen and poor vascular supply significantly hinder the penetration of urate-lowering therapies into the core of the tophus and restrict the infiltration of immune cells such as macrophages. These limitations impede crystal clearance and resolution of inflammation, contributing to the tendency of tophi to persist and resist spontaneous regression (175). Importantly, the fibrous capsule is not an inert structure. As an active fibrotic interface, it contributes directly to tissue damage by degrading cartilage and bone matrix through mechanical compression and the secretion of MMPs produced by myofibroblasts. In combination with osteoclast activation induced by urate crystals, these processes lead to progressive destruction of periarticular soft tissue, joint deformity, and the characteristic “punched-out” bone erosions observed in late-stage gouty arthritis (176). Together, these processes establish the fibrous capsule as both a protective barrier and a driver of chronic pathology. Its persistence sustains inflammation, limits therapeutic access, and accelerates structural joint damage, making targeted modulation of fibrotic pathways a critical priority for improving long-term outcomes in chronic gout.

### 8.2 Relative stability and dynamic equilibrium of the tophi microenvironment

Mature tophi function as relatively independent immune microenvironments, defined by distinctive physicochemical characteristics such as low pH and hypoxia. These conditions profoundly influence sodium urate solubility, immune cell activity, and therapeutic drug efficacy (177). This specialized microenvironment not only stabilizes urate crystals but also shapes the phenotype and function of resident cells. Single-cell transcriptomic studies reveal that macrophages in the tophus corona characterized by SPP1, MMP9, and CHI3L1 expression possess dual roles in immune modulation and matrix remodeling. These macrophages can differentiate into osteoclasts and communicate with matrix cells through integrin-associated signaling pathways, thereby contributing to the maintenance of tophus structure. In parallel, T-cell populations within tophi shift from inflammatory phenotypes to more immunoregulatory subtypes, reflecting a transition from a predominantly proinflammatory environment to a relatively suppressive one (48). The persistence and gradual enlargement of tophi depend on a dynamic equilibrium between ongoing crystal deposition in the core and the development of the surrounding fibrous capsule. This encapsulating structure acts as a physical barrier that isolates crystals from adjacent tissues. The specialized macrophages noted above participate in extracellular matrix remodeling by secreting matrix metalloproteinases, which regulate capsule formation and influence local tissue metabolism (45, 177). Even when episodes of acute inflammation occur, they tend to remain localized within or around the fibrous capsule and are typically insufficient to eliminate the central urate mass. Evidence from zebrafish models shows that persistent crystal accumulation induces macrophage-driven granuloma-like structures, closely resembling chronic tophus progression in humans (178). However, this delicate equilibrium can be disrupted by various factors capable of initiating acute inflammatory flares. A rapid reduction in serum uric acid during urate-lowering therapy can destabilize crystal surfaces and trigger the release of proinflammatory substances. Elevated levels of α1-antitrypsin can also inhibit neutrophil elastase–mediated degradation of IL-1β and IL-6, leading to recurrent inflammation (103). In addition, in patients with long-standing disease or advanced age, mixed deposition of calcium pyrophosphate dihydrate crystals may alter dissolution kinetics and further destabilize the tophus environment (45). In summary, the stability of urate crystals, the functional state of resident immune and stromal cells, and the integrity of the peripheral fibrous capsule interact in a finely balanced manner. The maintenance or disruption of this balance directly influences tophus progression and the occurrence of acute gout flares.

## 9 Potential therapeutic strategies for tophi

As a defining feature of advanced gout, tophus formation reflects a complex pathophysiological process driven by the combined influences of MSU crystal deposition, chronic inflammation, and fibrotic tissue remodeling. The trajectory of disease progression is shaped by both endogenous regulatory pathways and genetic determinants. A deeper understanding of these mechanisms and the identification of therapeutic targets are crucial for improving clinical outcomes in patients with chronic gout (179, 180). Uric acid supersaturation is the fundamental prerequisite for MSU crystal formation, and insufficient urate excretion remains the predominant cause of hyperuricemia and subsequent crystal deposition in most populations. Endogenous molecular pathways play central roles in regulating tophus development. The NLRP3 inflammasome is a key mediator of MSU crystal–induced inflammatory responses, and IL-1β serves as a principal downstream cytokine that amplifies inflammation. Although current literature provides limited detail on pathways such as A20, SIRT1, and Nrf2, available evidence suggests that any endogenous mechanism capable of suppressing NLRP3 activation, reducing NETosis, or mitigating fibrosis has the potential to delay the transition from acute gouty inflammation to chronic tophaceous lesions (181–183).

Genetic background is another important determinant in the progression from hyperuricemia to tophaceous gout. Polymorphisms in genes involved in urate transport and inflammatory signaling have become a major focus of recent research. The ABCG2 rs2231142 T allele has been strongly associated with early-onset gout, with significantly higher frequency in early-onset cohorts compared with late-onset individuals. A multi-cohort meta-analysis reported an odds ratio of 1.60 for this variant, suggesting that it may impair renal or intestinal urate excretion, thereby increasing both hyperuricemia risk and susceptibility to tophus formation (184, 185). SLC2A9 and URAT1 are key transporters that regulate uric acid reabsorption and excretion; abnormalities in their structure and function, genetic variants, and regulatory mechanisms play a significant role in gout. During acute gout attacks, urate crystals activate inflammatory pathways; in contrast, drugs such as allopurinol reduce both serum UA and inflammation by downregulating URAT1/GLUT9 and inhibiting the IL-1α/TLR4 pathway (186). Furthermore, the secretory function of intestinal ABCG2, in conjunction with renal transporters, helps maintain uric acid homeostasis; GLUT9 is also expressed in the intestine and participates in excretion (187). Additionally, environmental triggers such as alcohol, temperature, and diet synergize with genetic susceptibility to jointly drive gout onset (188). Genetic variations are the primary congenital risk factors for gout. Abnormalities in uric acid transport genes such as SLC2A9, SLC22A12, and ABCG2 lead to increased renal reabsorption of uric acid and impaired excretion, laying the groundwork for hyperuricemia. External environmental factors can interact synergistically with these genetic defects. A high-purine diet, alcohol consumption, and high fructose intake increase uric acid synthesis, while metabolic disorders and insulin resistance further disrupt the homeostasis of uric acid metabolism. Concurrently, unhealthy lifestyle habits such as prolonged sitting and irregular sleep patterns can induce low-grade systemic inflammation. This interacts with the inflammatory pathway triggered by urate crystals, jointly promoting the progression of hyperuricemia to gout, exacerbating joint damage, and contributing to the chronicity of the condition, thereby influencing the course of gout development. Although polymorphisms in genes such as NLRP3 and IL1B have not yet been comprehensively investigated, their central roles in mediating the inflammatory response to MSU crystals imply that genetic variations in these pathways may modulate the threshold at which asymptomatic hyperuricemia progresses to clinically significant tophus formation, by regulating the magnitude and duration of inflammatory responses.

Systems biology and genome-wide association studies (GWAS) have opened new avenues for identifying key regulatory factors of tophi. GWAS has identified multiple common and rare variant loci in genes such as SLC2A9, SLC22A12, and ABCG2. Among these, variants in SLC2A9 are significantly associated with serum uric acid levels, and heterogeneity exists across different populations (189). Studies have found that in patients with uric acid stones, miR-143-3p and miR-4770 may participate in stone formation by regulating the expression of SLC2A9 and SLC22A12 (190). By integrating genomic, transcriptomic, and proteomic datasets, systems biology approaches enable discrete genetic signals to be contextualized within broader molecular networks, including pathways regulating uric acid metabolism, inflammation, and tissue remodeling. This integrative strategy offers considerable promise for uncovering central regulatory modules and molecular nodes that drive tophus development, thereby providing a foundation for designing new targeted therapies (191).

Targeted intervention strategies have achieved incremental progress across the different stages of tophus formation. During the crystal nucleation stage, novel uricase-based therapies have shown substantial efficacy. Polyethylene glycol–modified urate oxidase converts uric acid into the more soluble allantoin, significantly promoting tophus reduction, although immunogenicity and related adverse reactions still require optimization (192). Vitamin C inhibits crystal aggregation by enhancing uric acid solubility, offering insights for molecular chaperone drug development (193), while URAT1 inhibitors indirectly reduce crystal deposition by promoting uric acid excretion (194). During the inflammatory and fibrotic stages of tophus progression, the NLRP3 inflammasome, neutrophil infiltration and NETosis, TGF-β signaling, and other profibrotic pathways have emerged as key therapeutic targets. IL-37 has shown potential in simultaneously suppressing inflammation and enhancing crystal clearance by modulating macrophage phenotype, presenting a promising direction for targeted drug development (195, 196). Comprehensive management of tophi requires sustained, effective urate-lowering therapy, with serum urate levels maintained below 0.30 mmol/L to reduce overall crystal burden (197). Adjunctive use of low-dose colchicine or similar agents can help mitigate chronic inflammation and soften the fibrotic capsule surrounding tophi (198). In terms of lowering uric acid levels, uric acid production inhibitors are represented by allopurinol and febuxostat. As a classic first-line medication, allopurinol blocks uric acid production by inhibiting xanthine oxidase, It offers a favorable cost-effectiveness ratio and is suitable for patients with hyperuricemia due to overproduction and those with concomitant renal insufficiency. However, Asian patients require screening for the HLA-B5801 gene to avoid severe allergic reactions, and dosing must be titrated starting from a low dose; Febuxostat is a highly selective xanthine oxidase inhibitor with a stronger uric acid-lowering effect than allopurinol. It is primarily metabolized by the liver and has minimal impact on renal function. It is suitable for patients who are allergic to or intolerant of allopurinol, as well as those with mild to moderate renal impairment. Caution is required regarding the risks of use in patients with cardiovascular disease, and low-dose colchicine may be co-administered at the start of treatment to prevent acute attacks. Uric acid excretion-promoting drugs, with benzbromarone as the core agent, reduce uric acid reabsorption by inhibiting the URAT1 transporter in the renal tubules. They are suitable for patients with impaired uric acid excretion (accounting for 90% of those with hyperuricemia) and those with normal or mildly impaired renal function. During treatment, daily fluid intake must be ≥2000 ml, and urine alkalization is required to prevent kidney stones. These drugs are contraindicated in patients with severe renal failure or kidney stones. In clinical practice, guidelines recommend prioritizing uric acid production inhibitors. If efficacy is inadequate, these may be combined with uric acid excretion-promoting drugs; however, the concurrent use of two uric acid production inhibitors is prohibited. Treatment regimens should be optimized based on patient comorbidities (such as cardiovascular disease, diabetes, and renal insufficiency) and genetic profiling to achieve stratified prevention and control as well as personalized treatment, thereby enhancing the efficacy and safety of uric acid-lowering therapy. During the acute phase, treatment focuses on anti-inflammatory and analgesic management. Nonsteroidal anti-inflammatory drugs (NSAIDs) are the first-line choice, with glucocorticoids as a second-line option. Colchicine serves as the core medication, while biologics (primarily IL-1β inhibitors) are indicated for patients with contraindications or intolerance to conventional therapies. NLRP3 inflammasome inhibitors, IL-1β pathway antagonists, and colchicine can specifically block inflammatory cascades and are suitable for acute flare-ups or for managing concomitant inflammation, though the risk of infection must be weighed against therapeutic efficacy. For patients with complications such as mechanical obstruction or infection, local interventions such as arthroscopic debridement or surgical excision may be used as adjunctive treatments; a multidisciplinary treatment model supported by 3D reconstruction technology can further improve efficacy (199). Dual-energy CT (DECT), as a crucial imaging modality in the diagnosis and treatment of gout, has enhanced the clinical diagnostic and efficacy evaluation systems. In the assessment of tophi, DECT enables accurate quantification of tophi, clearly displaying their size, distribution, and relationship with surrounding tissues, outperforming traditional imaging modalities. Furthermore, DECT allows for dynamic monitoring of urate crystal dissolution and tophi reduction during treatment, enabling objective evaluation of the efficacy of urate-lowering and anti-inflammatory therapies. This facilitates timely adjustments to treatment regimens, providing imaging support for personalized gout management and further enhancing the precision and standardization of diagnosis and treatment (200). In the future, it will be necessary to continuously optimize the safety and efficacy of targeted drugs, refine personalized comprehensive treatment plans, and establish a personalized prevention and control system by integrating uric acid transporter gene polymorphisms, individual metabolic characteristics, and environmental risk factors. For example, in patients with URAT1 hyperactivity caused by SLC22A12 variants, URAT1 inhibitors should be the first-line choice; for patients with impaired uric acid excretion due to ABCG2 mutations, uric acid excretion-promoting drugs may be combined with xanthine oxidase inhibitors. Concurrently, by integrating individual metabolic characteristics and environmental factors, implement stratified prevention and control. For patients with high genetic susceptibility and concomitant metabolic abnormalities, intensify dietary interventions and combination drug therapy, and dynamically monitor serum uric acid levels and inflammatory markers. This approach enables precise screening, personalized treatment, and long-term management of gout, significantly enhancing prevention and treatment outcomes and providing a new breakthrough for the precise prevention and treatment of tophi.

## 10 Summary and outlook

**Figure 4:**
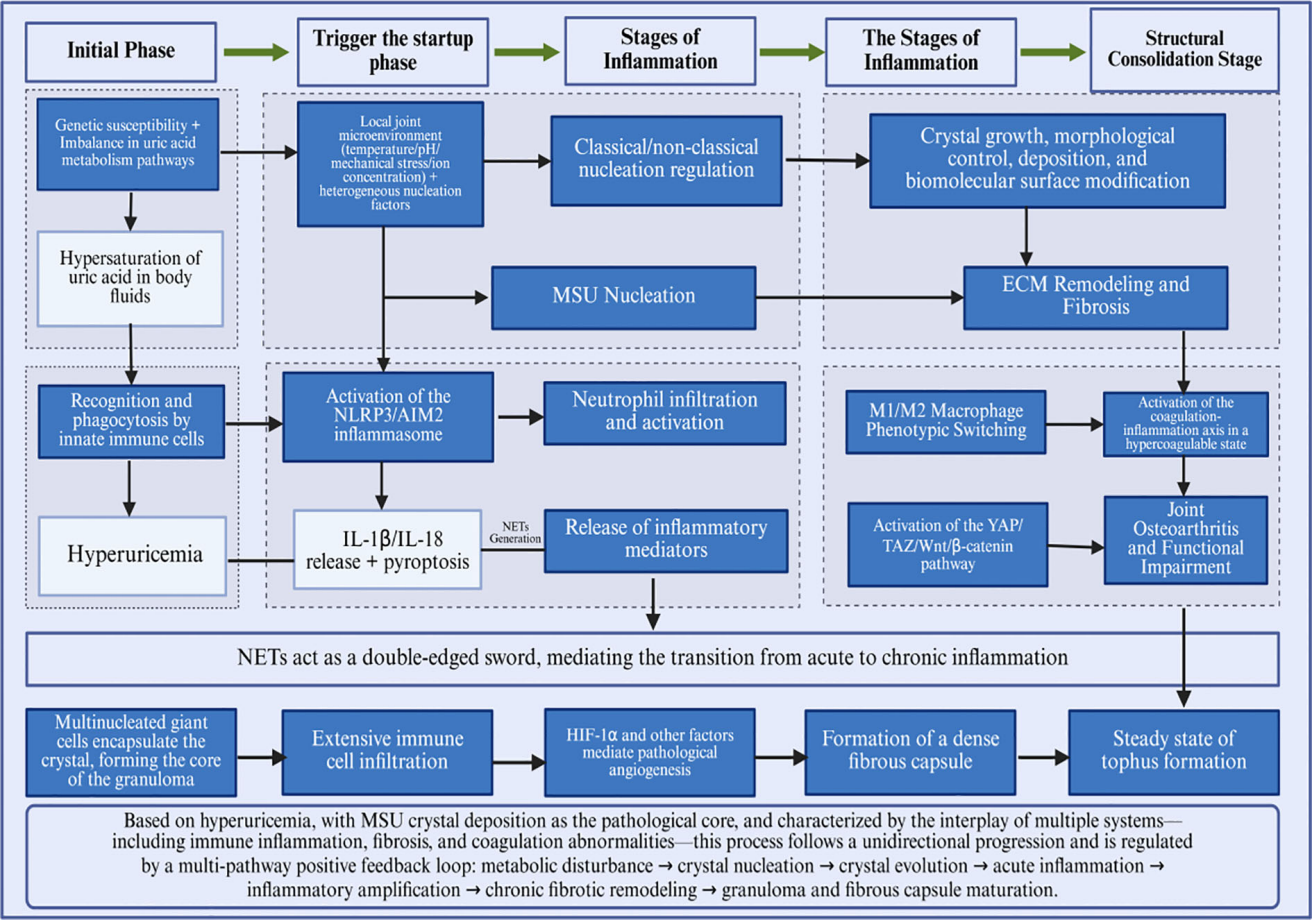
Schematic diagram of a multi-stage dynamic model of tophus formation. With hyperuricemia as the initiating factor, MSU crystal deposition as the pathological core, and the multi-system interaction of immune inflammation–fibrosis–coagulation abnormalities as the main pathway, the model follows a sequence of metabolic disorders → crystal nucleation → crystal evolution → acute inflammation → inflammatory amplification → chronic fibrotic remodeling → granuloma - and fibrous capsule maturation, elucidating the entire process of tophus formation from asymptomatic hyperuricemia to chronic structural lesions.

Tophus formation is a progressive and dynamic pathological process that begins with hyperuricemia as its fundamental basis and is driven forward by alterations in the local microenvironment. In the initiation stage, disruptions in uric acid production and excretion lead to systemic urate supersaturation. Local physicochemical factors such as reduced pH, lower temperature, mechanical stress, and the presence of diverse nucleation substrates collectively promote the nucleation of MSU crystals. During crystal formation and growth, both classical and non-classical nucleation mechanisms govern nucleation kinetics, crystal morphology, and aggregation patterns, while surrounding biomolecules and environmental cues further modulate crystal stability. Once formed, MSU crystals are recognized and internalized by innate immune cells, activating the NLRP3 inflammasome and inducing IL-1β release, which initiates acute inflammatory responses. Neutrophil infiltration and activation, along with the formation of NETs, intensify inflammation while simultaneously contributing to crystal sequestration and the transition from acute to chronic disease. As inflammation begins to resolve, macrophages undergo a shift from M1 to M2 phenotypes, fibroblasts become activated and initiate extracellular matrix remodeling, and coagulation abnormalities promote a hypercoagulable state. These processes collectively drive the progression toward chronic granulomatous inflammation. In the advanced phase, multinucleated giant cells, persistent immune cell infiltration, and pathological angiogenesis work together to construct mature tophi characterized by a dense fibrous capsule that stabilizes the local microenvironment (**Figure 4**). Current research has elucidated the central roles of urate metabolism, inflammatory regulation, and fibrosis-related pathways in this process, and has also identified genetic contributors, including polymorphisms in genes such as ABCG2, that influence susceptibility to tophus formation.

Although substantial progress has been made in elucidating the mechanisms underlying tophus formation, several critical gaps remain. The molecular processes that govern MSU crystal nucleation are still not fully defined, and the regulatory networks controlling NET formation and macrophage phenotypic transitions require more comprehensive characterization. In addition, the key molecular drivers of fibrous capsule development have yet to be clearly delineated, and the interplay between genetic susceptibility and environmental triggers remains insufficiently understood. Future research should employ advanced methodologies such as systems biology, single-cell transcriptomics, spatial omics, and high-resolution imaging to map the regulatory landscape of tophus formation with greater precision. These tools will help identify stage-specific molecular switches, core signaling hubs, and novel therapeutic targets across the initiation, amplification, and chronic remodeling phases of tophus development. There is also a need to refine therapeutic strategies. Precision treatment models that optimize the use of urate-lowering, anti-inflammatory, and anti-fibrotic agents will be essential for improving efficacy and minimizing adverse effects. Emerging therapeutic concepts, including targeted interventions aimed at enhancing crystal clearance, modulating the immune microenvironment, and disrupting fibrotic encapsulation, warrant exploration. Potential approaches include NET scavengers, TGF-β pathway inhibitors, and agents that modulate macrophage polarization. Overall, integrating genetic information, such as ABCG2 polymorphisms into personalized prevention and treatment frameworks will be crucial for establishing a stratified intervention model tailored to each pathological stage.
